# CAPS and Munc13: CATCHRs that SNARE Vesicles

**DOI:** 10.3389/fendo.2013.00187

**Published:** 2013-12-04

**Authors:** Declan J. James, Thomas F. J. Martin

**Affiliations:** ^1^Department of Biochemistry, University of Wisconsin, Madison, WI, USA

**Keywords:** CAPS (aka CADPS), Munc13, priming factors, vesicle fusion, SNAREs, multi-subunit tethering complexes

## Abstract

CAPS (Calcium-dependent Activator Protein for Secretion, aka CADPS) and Munc13 (Mammalian Unc-13) proteins function to prime vesicles for Ca^2+^-triggered exocytosis in neurons and neuroendocrine cells. CAPS and Munc13 proteins contain conserved C-terminal domains that promote the assembly of SNARE complexes for vesicle priming. Similarities of the C-terminal domains of CAPS/Munc13 proteins with Complex Associated with Tethering Containing Helical Rods domains in multi-subunit tethering complexes (MTCs) have been reported. MTCs coordinate multiple interactions for SNARE complex assembly at constitutive membrane fusion steps. We review aspects of these diverse tethering and priming factors to identify common operating principles.

## Trafficking in the Secretory and Endosomal Pathways

The transport of proteins and membranes in the secretory pathway is vectorial with vesicle formation in a donor compartment coupled to vesicle transport and subsequent fusion in an acceptor compartment. Vesicle delivery to an acceptor membrane involves several layers of interaction that confer targeting specificity involving tethering, docking, and priming of vesicles. These lead to SNARE pairing that mediates fusion of the vesicle with the acceptor membrane. In exocytic vesicle fusion with the plasma membrane, as well as for intracellular membrane fusion events, a diverse set of accessory (tethering and priming) factors are required to prime vesicles for fusion. Accessory factors commonly interact with vesicle and target membrane constituents that are characteristic of a membrane compartment such as Rab proteins (1, 2) and phosphoinositides (3, 4). Accessory factors also interact with SNARE proteins to promote SNARE protein complex assembly usually in association with proteins of the Sec1/Munc18 (SM) family. Accessory factors for constitutive trafficking include the tethering factor complexes Dsl1 for Golgi to ER transport, HOPS for late endosome fusion, and exocyst for exocytic fusion. Accessory factors for regulated vesicle exocytosis include the priming factors CAPS and Munc13. These exhibit sequence and structural similarity with tethering factor subunits (e.g., exocyst Sec6) (5, 6), which suggests there may be common features for these diverse accessory factors. We review aspects of tethering and priming factor function at several trafficking stations attempting to identify common operating principles.

## SNARE Proteins in Membrane Fusion

Biochemical reconstitution studies of membrane trafficking in the Golgi led to the discovery of SNARE (soluble *N*-ethylmaleimide sensitive factor attachment protein receptor) proteins as the general machinery for membrane fusion (7). The initial identification of neuronal syntaxin-1, SNAP-25, and VAMP2 (aka synaptobrevin2) as SNARE proteins in brain membrane extracts prompted advances for understanding Ca^2+^-triggered vesicle fusion events for neurotransmitter and peptide secretion (8, 9). An essential role for neuronal SNAREs in regulated vesicle exocytosis was indicated by finding them to be the substrates for the *Clostridial* zinc endopeptidase toxins (10–14). The further characterization of proteins in the SNARE protein superfamily generally facilitated research on membrane trafficking throughout the secretory and endosomal pathways (15). Much current research in membrane fusion is focused on understanding how accessory factors prime vesicles for fusion by regulating SNARE complex assembly.

SNARE proteins, usually C-terminal tail-anchored membrane proteins with membrane-proximal helical SNARE motifs, are grouped into syntaxin, SNAP-25, and VAMP families based on sequence relatedness, and referred to as “Q” or “R” SNAREs based on highly conserved glutamine or arginine residues in the zero layer of the SNARE motif (e.g., syntaxin-1 as Qa-, SNAP-25 as Qbc-, VAMP2 as R-SNARE) (16). The reconstitution of SNARE proteins into liposomes demonstrated that SNAREs are sufficient for mediating membrane fusion (17). Although the details of how SNARE proteins fuse membranes is emerging, structural, and biochemical analyses indicate that SNARE proteins present on two apposed membranes form a trans-SNARE complex to pin membranes close together (18, 19). Membrane fusion ensues when trans-SNARE complexes zipper-up through coiled-coil interactions in helical SNARE motifs (16, 20). The formation of a tight coiled-coil bundle, characteristically containing 3Q (Qa, Qb and Qc, or Qa and Qbc) and 1R SNARE motifs, is coupled to membrane fusion events throughout the secretory pathway (21). For regulated vesicle exocytosis, SNARE complex assembly is thought to proceed by a two-stage process with the initial formation of heterodimeric QaQbc complexes of plasma membrane syntaxin-1 with SNAP-25 followed by the insertion of the vesicle R-SNARE VAMP2 to form heterotrimeric (RQaQbc) SNARE complexes (8). Alternative assembly pathways have been suggested (22–25).

At least 44 SNARE protein isoforms in vertebrate cells are distributed throughout membrane trafficking pathways (15). It was proposed that unique cognate SNARE pairing contributes to specificity for vesicle targeting to acceptor membranes (26, 27). However, it has been noted that SNARE pairing can be promiscuous and may not be the sole determinant of vesicle targeting specificity (28, 29). Targeting specificity is likely combinatorial consisting of multiple levels of interaction requiring accessory factors that are recruited to membranes by interactions with Rabs, phosphoinositides, and SNAREs (26). Accessory factors acting with SM proteins promote stages of SNARE complex assembly and enable specific SNARE pairing for fusion. For example, recent studies revealed differential effects of the SM proteins Munc18-1 and Munc18c for enabling fusion on cognate but not on non-cognate SNARE proteins (30, 31).

## SM Proteins in Membrane Fusion

The SM (Sec1/Munc18) protein family consists of soluble proteins that are required for membrane trafficking (32–34). SM proteins are grouped into four highly conserved subfamilies across eukaryotes. In spite of their sequence homology, SM proteins encode a high degree of specificity for SNARE protein interactions. A common theme is SM protein-directed interaction with Q-SNAREs at exocytic (Munc18/Sec1), ER-Golgi (Sly1), endosomal-lysosomal (Vps33), and endosomal (Vps45) membrane trafficking stations (32, 35–38). The mode of Q-SNARE-binding by different SM proteins appears to differ but the SM proteins may generally function in stabilizing SNARE protein complexes (39). Studies on the interaction of the neuronal SM protein Munc18-1 with the Qa-SNARE syntaxin-1 have played a prominent role in understanding SM protein function even though Munc18-1 has unique features that distinguish it from other SM proteins (32). Munc18-1 chaperones syntaxin-1 to the plasma membrane (32, 39). At the plasma membrane, Munc18-1 stabilizes a closed form of syntaxin-1, which is unable to form heterodimeric complexes with the Qbc-SNARE SNAP-25 (40). The closed configuration of syntaxin-1 may prevent unwanted interactions with SNARE proteins as the complex traffics to the plasma membrane (41). Eliminating Munc18-1 reduces syntaxin-1 delivery to the plasma membrane, abrogates dense-core vesicle (DCV) docking, and abolishes triggered exocytosis (42).

Recent findings that Munc18-1 accelerates SNARE-catalyzed liposome fusion help to reconcile the role of Munc18-1 as a chaperone with its essential role in regulated exocytosis (30). Munc18-1 stimulates trans-SNARE complex formation and membrane fusion but does so by switching from an inhibitory to a stimulatory mode. The switch from an inhibitory to a stimulatory mode in liposome fusion for Munc18-1 requires pre-incubation with both R- and Q- SNARE proteins (VAMP2 and syntaxin-1/SNAP-25) (43), which suggests that Munc18-1 utilizes specific interaction sites on the SNARE proteins. Recent studies have identified some of these sites on VAMP2 (C-terminal) and syntaxin-1 (N- and C-terminal) (30, 31). Interestingly, Munc18c appeared to operate differently on its cognate SNAREs lacking an inhibitory mode on syntaxin-4. In addition, recognition sites on shared cognate VAMP2 differed with C-terminal sites for Munc18-1 and N-terminal sites for Munc18c (30, 31). While both Munc18-1 and Munc18c promote the assembly of cognate SNARE protein complexes, they appear to do so by distinct sets of interactions that play a role in establishing specific SNARE protein pairing.

Tethering and priming factors act with SM proteins to promote SNARE complex assembly. Munc13-1 is proposed to enhance the switching of Munc18-1 from an inhibitory to stimulatory mode for regulated vesicle exocytosis (44) as discussed below. At other fusion events, cognate SM proteins also function in concert with accessory factors. In vacuolar fusion, the SM protein Vps33 operates as a subunit of a HOPS (homotypic fusion and vacuole protein sorting) complex, a multi-subunit tethering complex that promotes trans-SNARE complex assembly (45). At other trafficking stations where SM proteins are not formally part of complexes, it is likely that tethering and priming factors cooperate with SM proteins in the assembly of SNARE complexes as noted below.

## Tethering Factors Integrate SNARE Protein Function for Constitutive Fusion

Tethering is considered to be a long-range interaction of a vesicle near a target membrane independent of cytoskeletal anchoring. Tethering factors may also operate to bring vesicles into closer proximity for trans-SNARE complex assembly (docking). Tethering factors have been classified as either long coiled-coil proteins or multi-subunit tethering complexes (MTCs). Several MTCs that function at distinct membrane trafficking steps have been identified (46) where they function as important interfaces between Rabs, phosphoinositides, and SNARE proteins (47). Tethering factors are very diverse but sequence comparisons indicate a subtle relatedness among subunits of a subset of MTCs in predicted coiled coils (48). Structural analysis of several MTC subunits from COG, Dsl, exocyst, and GARP complexes indicate a homologous tertiary structure composed of an extended rod-like domain made up of helical bundles (49, 50). MTCs with this structural signature were termed members of a Complex Associated with Tethering Containing Helical Rods (CATCHR) family (50). The CATCHR homology is not restricted to MTC subunits and is found in Myo2p (Myosin V) and in CAPS and Munc13 proteins (see below) (5). In the following, we discuss a well-studied MTC (HOPS) whose subunits lacks CATCHR homology followed by two other well-studied MTCs (exocyst and Dsl1p) that contain subunits with CATCHR homology.

**Figure 1:**
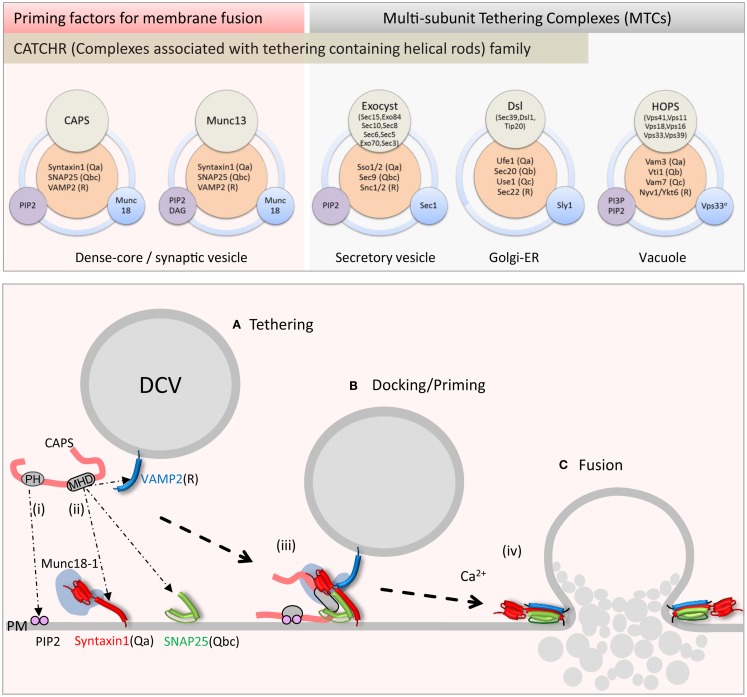
**Upper panel: schematic of priming factor (CAPS, Munc13) and tethering complex (exocyst, Dsl1, HOPS) composition and interactions**. Multi-domain CAPS and Munc13 proteins interact with neuronal SNAREs and PIP_2_ and co-function with the SM Munc18-1. The exocyst complex with eight subunits interacts with cognate SNAREs, PIP_2_, and the SM Sec1. The Dsl1 complex with three subunits interacts with cognate SNAREs and co-functions with the SM protein Sly1. The HOPS complex with six subunits interacts with cognate SNAREs, PI3P, and PIP_2_, and contains the SM protein Vps33. Lower panel: schematic of CAPS function in vesicle priming depicting **(i)** PH domain binding to PIP_2_ and **(ii)** MHD1-mediated SNARE binding. Simultaneous binding of vesicle and plasma membrane constituents by CAPS might tether vesicles **(A)** in proximity to the SNARE proteins. These interactions could lead to **(iii)** syntaxin-1/SNAP-25 heterodimer formation followed by VAMP2 insertion to form trans-SNARE complexes in priming **(B)**. **(iv)** Full SNARE complex zippering in response to elevations of intracellular calcium mediated by synaptotagmin and complexin triggers vesicle fusion **(C)** and contents release into the extracellular space.

The HOPS complex is an MTC of ∼663 kDa comprised of six subunits Vps41, Vps11, Vps18, Vps16, Vps33, and Vps39 (Figure 1, upper) (45). Conserved across eukaryotes, HOPS was initially identified for its role in yeast vacuole fusion, which is a correlate of endo-lysosomal fusion in vertebrate cells (48). The HOPS complex interacts with lipids, Rabs, and SNARE proteins (45). As a complex, HOPS mediates vesicle tethering as well as membrane fusion (51). Ultra-structural analysis of purified HOPS by EM-reconstruction techniques revealed that the holo-complex is a 30 nm long molecular monolith (52, 53). A combination of biochemical and structural reconstruction indicated that its Rab-interacting subunits Vps41 and Vps39 are positioned at either end of the complex (53). This arrangement of Rab-binding domains may mediate HOPS-dependent vesicle tethering. The SM protein Vps33 and nearby Rab-binding Vps39 are juxtaposed toward the membrane where HOPS may couple vesicle tethering with SNARE complex assembly. The monolith-like HOPS would stand like Stonehenge coordinating vacuole–vacuole fusion sites arranged around a vertex ring (45). Fusion between large organelles such as vacuoles likely requires solid tethering foundations provided by the HOPS complex for coordinating large surface area membrane fusion events. HOPS also requires the phosphoinositides PI3P and PI(4,5)P_2_ to coordinate its function (45). Overall, HOPS promotes the formation of a 3Q:1R trans-SNARE complex consisting of Vam3(Qa), Vti1(Qb), Vam7(Qc), and Nyv1(R) (54). Besides the requirement of the SM protein subunit Vps33 in SNARE complex formation, Vps16 and Vps18 interact with the soluble Qc-SNARE Vam7 and mediate a rate-limiting step for Qc-SNARE entry into a fusion-competent SNARE complex (55). The SM protein subunit Vps33 also interacts with the SNARE-binding Vps16 subunit (56), which may together with Vps18 form a SNARE-binding interface for the HOPS complex. The HOPS complex lacks the CATCHR domain homology but is an example of an MTC that integrates Rab, phosphoinositide, and SNARE interactions for tethering and compartment-specific SNARE complex assembly.

The exocyst complex, originally discovered in yeast, is a conserved multi-subunit complex ∼750 kDa composed of eight subunits Sec3, Sec5, Sec6, Sec8, Sec10, Sec15, Exo70, and Exo84 (Figure 1, upper) (57). Exocyst functions in polarized constitutive exocytosis in budding yeast, plants, and vertebrate cells, and is thought to tether vesicles to exocytic sites at the plasma membrane. Ablation of exocyst components in yeast results in the mis-localization of the complex and an accumulation of vesicles within the cell interior. At the plasma membrane, the exocyst is an octomeric holo-complex but the exact pathway for assembly of the complex is under active study. Studies in yeast suggest that vesicle tethering is achieved by complex assembly initiated between vesicle-bound Sec4 (Rab)-Sec15 and plasma membrane-targeted Exo70 and Sec3 subunits (58). The fully assembled exocyst complex localizes to growth cones in neurons where it plays an important role in membrane addition (59). The plasma membrane recruitment of exocyst is mediated through interactions of exocyst subunits Exo70 and Sec3 with GTPases (Rho and cdc42 family) and the phosphoinositide lipid PI(4,5)P_2_ (60, 61).

Structural studies indicate that Sec6, Sec15, Exo70, and Exo84 subunits contain homologous CATCHR domains. These CATCHR domains mediate inter-subunit interactions to provide an elongated structure for tethering as well as interactions with other proteins (GTPases) (62). Besides a role in tethering, exocyst subunits interact with SNARE and SM proteins to control SNARE complex assembly (57). The exocyst subunit Sec6, as a dimer, interacts with the Qbc-SNARE Sec9 and inhibits the formation of Qabc acceptor SNARE complexes (63). In addition, Sec6 interacts with the SM protein Sec1 when it is part of the exocyst complex mediated by N-terminal sites in Sec6, which functionally overlap with Sec9 binding sites (64). Truncation of these N-terminal sites in Sec6 (the CATCHR domain is C-terminal) inhibits dimerization as well as Sec9 and Sec1 binding. In contrast, mutations in the Sec6 CATCHR domain disrupt exocytosis and polarized localization of exocyst but not exocyst complex formation or Sec6-Sec9 interactions (64). It is not known whether these Sec6 CATCHR domain mutations impair exocyst-Sec1 interactions. Nevertheless, it appears that Sec6 mediates interactions that anchor the exocyst to sites of polarized exocytosis (65). One speculation is that Sec6-Sec9 interactions may help stage the assembly of a Qabc-SNARE complex concomitant with arrival of the vesicle containing other exocyst components to promote trans-SNARE complex formation mediated by exocyst-Sec1 interactions. Notably the formation of a Q-SNARE complex by exocyst is similar to the HOPS recruitment of Vam7. In summary, the exocyst complex tethers through vesicle Rab (Sec4) and plasma membrane phosphoinositide and GTPase interactions, and associates with the SM protein Sec1 and SNAREs to promote or stabilize SNARE complex assembly.

The Dsl1 complex is the smallest of the MTCs at ∼250 kDa and is composed of three subunits Dsl1, Tip20, and Sec39 (Figure 1, upper) (66, 67). As an essential protein complex in yeast, it is required for the fusion of Golgi-derived vesicles with the ER. Two of the three subunits have homologs in humans that are involved in retrograde trafficking pathways between the Golgi and ER (48). Structural studies of the yeast Dsl1 complex indicate that Sec39 and Tip20 subunits are bridged by Dsl1 through interactions of the CATCHR domain of Dsl1 with that of Tip20 to assemble a 20 nm structure (68). The Dsl1 subunit interacts with the COP1 coat complex and may serve as a direct link securing incoming Golgi-derived COP1 vesicles at the ER membrane (69). The Dsl1 complex interacts with ER resident SNAREs Ufe1(Qa), Sec20(Qb), and Use1(Qc) to stabilize a Qabc-SNARE complex (66, 70) in conjunction with the SM protein Sly1 (71). Interestingly, interactions with the Qb-SNARE Sec20 appear to be mediated by the CATCHR domain of the Tip20 subunit (68). The recruitment of the Qc-SNARE Use1 into SNARE complexes depends on Sec39 and Dsl1 subunits interacting with Tip20 (66). Sly1 interactions with Ufe1 and SNARE complexes are part of a larger complex that includes Dsl1 representing a complex that coordinates Golgi to ER retrograde traffic (38, 71). Overall, the Dsl1 complex links vesicle tethering to SNARE complex assembly and fusion at sites in the ER.

Tethering factors are essential coordinators linking vesicle arrival with SNARE complex assembly. Individual MTC subunits mediate a number of protein and phospholipid interactions. CATCHR domains in MTC subunits that possess them appear to generally mediate interactions with other subunits or with other proteins including SNAREs. One common theme illustrated by tethering factors at several vesicle trafficking stations is the on-demand assembly of Q-SNARE complexes with vesicle arrival. Recent studies (29) suggested that Qc-SNAREs are a particularly important determinant for selectivity in SNARE pairing for fusion. Thus, it is notable that an interaction specific for the Qc-SNARE is found for HOPS (55), and that exocyst and Dsl1 complexes bind Sec9 Qbc- or Use1p Qc- SNAREs, respectively. Overall this suggests an important role for tethering factors in promoting the entry of Qc-SNAREs into SNARE complexes to enable appropriately paired, compartment-specific fusion.

## Priming Factors Integrate SNARE Protein Function for Regulated Fusion

The release of neurotransmitters from synaptic vesicles (SVs) at the neuronal synapse or peptides from DCVs of neuroendocrine cells occurs by regulated exocytosis. Pools of SVs and DCVs are stored near the plasma membrane in various states prior to fusion (72). Traditionally, vesicles are viewed as progressing through states of tethering, docking, and priming prior to Ca^2+^-triggered fusion (73). Recent high-pressure freeze EM (74) and fluorescence microscopy mobility studies of vesicles (75) indicate that docking and priming may be closely linked steps. Physiological and genetic studies suggest that SNARE complex assembly occurs during priming (76, 77). SV exocytosis is strongly inhibited in mice lacking Munc13-1 (78, 79) and CAPS (80). DCV exocytosis is also strongly impaired in cells lacking Munc13-1 or CAPS (78, 81–83). Thus, major accessory factors for SV and DCV priming are the related CAPS and Munc13 proteins. Munc18-1 is also involved in SV and DCV priming but its role in priming in cells is difficult to separate from its upstream role as a syntaxin-1 chaperone that influences vesicle docking (42, 84). It is likely that CAPS and Munc13 proteins co-function with Munc18-1 in vesicle priming.

**Figure 2:**
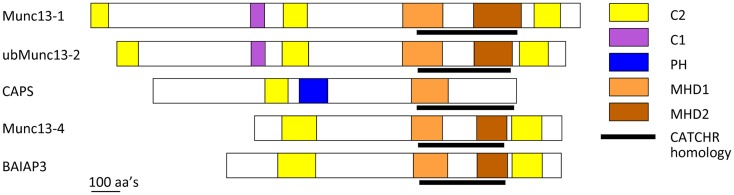
**Schematic of CAPS/Munc13 family of priming factors**. Colored boxes indicate relative location of C2, C1, PH, MHD1, and MHD2 domains. The CATCHR homology region is indicated by black line.

CAPS and Munc13 proteins are related in sequence (Figure 2) (6, 85). CAPS and Munc13-1 exhibit ∼40% sequence similarity in a C-terminal region that contains the MHD1 homology domain. All Munc13 proteins (including Munc13-4 and BAIAP3) but not CAPS proteins also share a more C-terminal MHD2 domain. Overall the C-terminal region of CAPS and Munc13 proteins exhibits weak sequence homology to the CATCHR domain of exocyst, COG, GARP, and Dsl1 complex subunits (Figure 2) (6). More convincingly, crystallographic studies of a Munc13-1(1148–1531) protein indicated strong structural similarity to the CATCHR region of the Sec6 subunit of the exocyst complex (5). This homology across diverse proteins implies an evolutionary relatedness but could also indicate a conserved functional role for the CATCHR domain. In MTCs, an inherent structural role for CATCHR domains was seen to be adapted to mediate protein–protein interactions that include SNARE-binding (50). Studies of the CAPS and Munc13 proteins indicate a role for this region in scaffolding SNARE proteins as well as for other protein interactions. In the following, we discuss membrane- and SNARE-binding features of CAPS and Munc13 proteins that have counterparts in MTCs.

## Priming Factors for Regulated Vesicle Exocytosis: CAPS

CAPS (aka CADPS) was discovered for its activity in regulating DCV exocytosis in neuroendocrine cells (86) and was found to correspond to the *Caenorhabditis elegans* UNC31 protein (87). Unc-31 deletion mutants exhibit a strong loss of DCV exocytosis and neuropeptide secretion (88–90) with moderate reductions in SV exocytosis and synaptic transmission (88, 89, 91, 92), which matches the conditional uncoordinated phenotype. By contrast, the phenotypes for *C. elegans* UNC13 mutants are much more severe. Unc-13 hypomorphs are paralyzed, and exhibit a strong loss of synaptic transmission with lesser impact on DCV exocytosis and neuropeptide secretion (89, 93, 94). Thus, *C. elegans* CAPS/UNC31 is essential for DCV exocytosis whereas Munc13/UNC13 plays a dominant role in SV exocytosis. In vertebrates, the requirements for vesicle priming are more complex in requiring both CAPS and Munc13 proteins. Vertebrates possess two CAPS genes (CAPS/CADPS and CAPS2/CADPS2) that control DCV exocytosis in chromaffin cells, pancreatic β cells, and neurons (83, 95–100). Munc13 proteins are encoded by five genes (Munc13-1, -2, -3, -4, BAIAP3). Munc13-1 is required for DCV exocytosis in pancreatic β cells (101, 102), which indicates that both CAPS and Munc13-1 are required for regulated insulin secretion. CAPS localizes to DCVs but not SVs in brain tissue (103). In spite of this, studies indicate that CAPS-1/2 KO mice exhibit as complete loss of synaptic transmission as reported for Munc13-1 KO mice (78, 80). Thus, it appears that CAPS and Munc13 proteins are both required for SV and DCV priming in vertebrate nervous and endocrine systems.

Recent studies have revealed CAPS to be a regulator of SNARE complex assembly. Attempts to detect direct CAPS interactions with soluble SNARE proteins were of limited success indicating only very low affinity interactions; however, recent studies revealed direct CAPS interactions with membrane-associated SNARE proteins (Figure 1, lower) (104–107). Liposomes containing syntaxin-1/SNAP-25 heterodimers or VAMP2 were found to retain CAPS in liposome flotation studies (104). CAPS interacted independently with either syntaxin-1 or SNAP-25 suggesting that CAPS might promote QaQbc-SNARE heterodimer formation. CAPS binding to syntaxin-1 was mediated by the membrane-proximal C-terminal SNARE motif (H3) and membrane linker domain sequences of syntaxin-1 (104). CAPS interactions with N-terminal regions of the SNARE motif of VAMP2 were also detected, which suggests that CAPS might recruit VAMP2 into syntaxin-1/SNAP-25 heterodimers for RQaQbc-SNARE complex assembly. As a SNARE-binding protein, CAPS stimulated the formation of SNARE complexes on liposomes (106) and promoted VAMP2 liposome docking on supported bilayer membranes containing syntaxin-1/SNAP-25 heterodimers indicating that stable trans-SNARE complex formation had occurred. These studies utilized pre-formed syntaxin-1/SNAP-25 heterodimers, and indicated that CAPS could promote VAMP2 insertion into QaQbc-SNARE heterodimers to assemble heterotrimeric SNARE complexes (Figure 1, lower). The activity of CAPS in promoting SNARE complex formation was also evident in studies of SNARE-dependent liposome fusion where CAPS markedly increased the rate and extent of fusion between donor VAMP2 liposomes and syntaxin-1/SNAP-25 acceptor liposomes (105, 106). These results also suggested that CAPS acts to promote the insertion of the R-SNARE VAMP2 into Qabc-SNARE syntaxin-1/SNAP-25 acceptors (Figure 1, lower) but it is not yet known if CAPS utilizes direct interactions with syntaxin-1/SNAP-25, with VAMP2, or with both to enable SNARE complex assembly. Additional studies are needed to determine the detailed mechanism of how CAPS enhances SNARE complex assembly.

A key issue is which CAPS domains mediate SNARE protein binding and SNARE complex assembly. The C-terminal region of CAPS/Munc13 proteins contains numerous α-helices, which makes standard recombinant protein analysis or yeast two-hybrid interaction studies very challenging (5, 108). We produced a set of recombinant proteins across the CAPS sequence based on secondary structure predictions and proteolysis studies, and tested these proteins for moderate-to-high affinity (0.2 μM) SNARE-binding (107). Only one protein fragment corresponding to rat CAPS(859-1073) was retained in flotation studies by syntaxin-1/SNAP-25 liposomes (107). CAPS(859-1073), which brackets the MHD1 homology region (Figure 2), exhibited submicromolar binding affinity for SNARE proteins and effectively competed with full-length CAPS(1-1289) for binding indicating that this region contains the major SNARE-binding domain of CAPS. Further studies with protein fragments suggested that the major SNARE-binding segment may consist of a helix in the center of MHD1 that contains a VAMP2 homology region. This corresponds to the N-terminal helix of the CATCHR homology region (Figure 2) (5, 6, 107). These studies did not exclude the possibility that other helices within the CATCHR homology domain provide additional lower affinity SNARE-binding. Thus, this region consisting of stacked α-helices could function as a scaffold to organize helical SNARE motifs. A recent report suggested that syntaxin-1 binding by CAPS was mediated by more C-terminal sequences within CATCHR; however, these studies employed constructs in yeast two-hybrid studies that may have encoded unfolded proteins (109). Studies on CAPS are consistent with a SNARE scaffolding role for the CATCHR homology region. However, other CAPS-protein interactions have also been reported for this region (110), which could indicate a more general role for the CATCHR domain as a protein interaction domain.

For MTCs, numerous membrane interactions can be achieved by multiple subunits. As a large multi-domain protein, CAPS may instead utilize multiple domains to mediate protein and lipid interactions. CAPS exhibits low affinity but functionally significant interactions with plasma membrane PIP_2_ via its central PH (pleckstrin homology) domain (82, 111). PIP_2_ enhanced CAPS stimulation of SNARE-dependent liposome fusion with wild-type but not with mutant PH domain CAPS proteins (105). Inclusion of PIP_2_ in the syntaxin-1/SNAP-25-containing acceptor liposomes was much more effective than inclusion in the VAMP2-containing donor membranes, which suggests that PIP_2_ is an important co-factor for CAPS in acting on plasma membrane SNARE proteins (Figure 1, lower). PIP_2_ may promote conformational or oligomerization changes in CAPS to enhance its SNARE interactions (111). In addition, because CAPS interacts with syntaxin-1 near its C-terminal linker that binds PIP_2_, this might allow CAPS to regulate the conformation of syntaxin-1 (104, 105). These results suggest a framework for understanding the actions of priming factors. CAPS utilizes two contacts with the membrane – one with membrane phospholipids via its PH domain and the other with SNAREs via its MHD1 domain – to promote the assembly of SNARE protein complexes.

CAPS localizes to DCVs (103, 112) and also interacts with plasma membrane PIP_2_, which could provide a trans-membrane interaction for vesicle tethering. However, such a tethering mechanism would likely be transient because of the low affinity PIP_2_ interactions. The basis for CAPS anchoring to DCVs via C-terminal interactions (112) remains to be clarified. Possible interactions with the DCV constituents phogrin (113), VMAT (114), ARF4/5 (115, 116), and RRP17 (110) have been suggested.

Tethering factors are thought to engage in long-range capture of vesicles (tethering) at the target membrane involving distances (>20 nm) at which SNARE complexes cannot assemble. MTC tethering complexes likely bring vesicles into closer proximity to enable SNARE complex formation and docking. CAPS functions in vesicle priming to promote the assembly of SNARE complexes that bridge vesicles to the plasma membrane, which may be expected to mediate vesicle docking. Indeed, *in vitro* studies of VAMP2 liposome docking onto syntaxin-1/SNAP-25-containing membranes revealed that CAPS could promote a stable docking complex (106). EM studies in *C. elegans* also indicated that CAPS/Unc31 was required for DCV docking to the plasma membrane (88). Although DCV docking defects were not reported for chromaffin cells from CAPS KO mice, this may be attributable to the small percentage of total vesicles that are primed in these cells (83).

## Priming Factors for Regulated Vesicle Exocytosis: Munc13

Munc13 proteins are thought to function in vesicle priming by interacting with SNARE proteins (44). Munc13-4, a short Munc13 isoform with N- and C-terminal C2 domains (C2A and C2B, respectively) bracketing the MHD1-MHD2 region (Figure 2), functions in the priming as well as the maturation of lysosome-related secretory granules for fusion in secretory cells of hematopoietic origin (117). Munc13-4 appears to function as a tether for granule-plasma membrane interactions mediated by vesicle-associated Rab27 (118, 119). Recent biochemical studies revealed that Munc13-4 exhibits Ca^2+^-regulated SNARE interactions modulated by its C2A domain and Ca^2+^-dependent membrane interactions mediated by its C2B domain (120). Munc13-4 promoted the fusion of VAMP2 donor liposomes with syntaxin-1/SNAP-25 acceptor liposomes that was dependent on Ca^2+^ and Ca^2+^-binding residues in each C2 domain (120). These results indicated that Ca^2+^-activated Munc13-4 can function similarly to CAPS by promoting the recruitment of the R-SNARE VAMP2 into Qabc-SNARE syntaxin-1/SNAP-25 acceptors for RQaQbc-SNARE complex assembly. The central MHD1-MHD2 region of Munc13-4 with CATCHR homology may mediate SNARE-binding but this has yet to be demonstrated. For Munc13-4 as for CAPS, it appeared that anchoring the protein to the membrane (via the Ca^2+^-dependent C2B domain) coupled to SNARE-binding was required to promote SNARE complex assembly and liposome fusion (120).

C-terminal regions of Munc13-1 were reported to interact with N-terminal domains of syntaxin-1 in yeast two-hybrid interaction studies (121, 122). Solution binding studies with recombinant Munc13-1 protein fragments localized N-terminal syntaxin-1 binding to Munc13-1(1181–1736), which corresponds to sequences beginning in MHD1 (Figure 2). This interaction was proposed to counteract Munc18-1-mediated stabilization of a “closed” form of syntaxin-1 to “open” it to enable syntaxin-1/SNAP-25 heterodimer formation. However, subsequent studies reported that Munc13-1(859–1531) (termed MUN domain) failed to interact with SNARE proteins in solution but did interact with liposome-integrated SNARE protein complexes (108, 123, 124). Munc13-1(859–1531) bound to syntaxin-1/SNAP-25 or syntaxin-1/SNAP-25/VAMP2 liposomes but not to syntaxin-1 liposomes, which suggested that Munc13-1 stabilizes SNARE complexes (123). Consistent with this, other studies provided evidence that Munc13-1(859–1531) stabilized parallel conformations of syntaxin-1/SNAP-25 heterodimers (124). Recent NMR studies with Munc13-1(859–1531) and soluble SNARE proteins indicated that Munc13-1(859–1531) interacts very weakly with the C-terminal SNARE domain of syntaxin-1 and with Munc18-1-bound syntaxin-1 (125). By contrast, a structurally defined Munc13-1(1148–1531) protein fragment exhibited further attenuated SNARE protein interactions possibly because this fragment lacked more N-terminal sequences (5).

In recent studies, a Munc13-1(529–1531) protein that contained C1 and C2B domains (Figure 2) was shown to operate on liposomal syntaxin-1-Munc18-1 to enable Ca^2+^-bound synaptotagmin C2AB to promote SNARE-dependent liposome fusion (126). It was proposed that the Munc13-1 fragment catalyzed a transition of Munc18-1-bound syntaxin-1 into syntaxin-1/SNAP-25 heterodimers to serve as acceptor complexes for VAMP2. This model for Munc13-1 action retains the feature of syntaxin-1 “opening” but proposes that the Munc13-1 C-terminal domain interacts with C-terminal rather than N-terminal regions of syntaxin-1 (126). In addition, these studies with a Munc13-1 fragment plus Munc18-1 suggested a possible exchange of the Qbc-SNARE SNAP-25 into Q-SNARE complexes as an important regulated step (126).

These studies suggest a mode of action for Munc13-1 in promoting the transition of closed syntaxin-1 monomers to syntaxin-1/SNAP-25 heterodimers. Similar effects for Munc13-4 and CAPS have not yet been demonstrated. Actual differences in the mechanisms of Munc13-1 and CAPS action on SNAREs could help to account for the non-redundancy of these factors for vesicle priming. However, future study will be needed to assess whether these apparent differences result from the use of different assays for CAPS and Munc13-1 proteins. Genetic studies in *C. elegans* have found that expression of an “open” syntaxin mutant by-passes DCV docking defects in CAPS/Unc-31 mutants (88) and SV docking defects in Unc-13 mutants (127) possibly indicating that both proteins enable a transition of syntaxin-1/Munc18-1 complexes to syntaxin-1/SNAP-25 complexes. Overall, the studies on Munc13-1 are compatible with a SNARE scaffolding role for the CATCHR homology region but the detailed mechanics of SNARE-binding remain to be worked out.

Munc13-1/2 proteins are also reported to exhibit Ca^2+^-dependent, high affinity PIP_2_ interactions via a central C2B domain (128). This interaction was significant for SV exocytosis in response to high frequency stimulation rather than for responses to single action potentials. The importance of C2B-mediated phosphoinositide interactions for DCV exocytosis in neuroendocrine cells has not been determined but could play a role in recruiting cytosolic Munc13-1/2 to sites of DCV exocytosis. The adjacent DAG-binding C1 domain of Munc13-1 mediates the membrane recruitment of Munc13-1 in response to DAG, however, a functional C1 domain in Munc13-1 is not required for Ca^2+^-stimulated vesicle exocytosis but rather for potentiated responses (129).

In contrast to the cytoplasmic localization of Munc13-1/2 in neuroendocrine cells, Munc13-1 localizes to the active zone in synapses where it associates with at least four other active zone proteins (RIM, bassoon, aczonin/piccolo, and CAST) via its N-terminal domain (130). This molecular complex likely serves a tethering role mediated by proteins anchored to both the presynaptic membrane and to SVs (130). Interactions with SVs may be mediated by a complex of RIM and Munc13-1 with Rab3 on the vesicle (131). Studies suggest that RIM activates Munc13-1 by converting it from an inactive dimer to active monomer (42). Standard chemical fixation methods had failed to reveal decreased SV docking in neurons from Munc13-1 KO mice, but high-pressure freezing techniques with EM tomography indicated that SVs were tethered but not docked in the absence of Munc13-1 (74). Similarly, high-pressure freezing followed by low-temperature fixation in *C. elegans* revealed a requirement for UNC13 in SV docking (88). Although the studies might suggest that priming and docking are functional and morphological aspects of the same process, more studies are needed in vertebrate neurons and endocrine cells where CAPS and Munc13 are co-required for vesicle priming.

## Summary

At multiple trafficking stations in secretory and endosomal pathways, diverse tethering and priming factors integrate multiple protein and lipid interactions to achieve compartment-specific SNARE complex assembly for fusion. The MTCs promote SNARE complex assembly by direct interactions of MTC subunits with Q-SNAREs and collaborative interactions with SM proteins. A subset of MTC subunits utilize structurally similar CATCHR domains to mediate inter-subunit interactions as well as SNARE protein interactions. At sites of regulated vesicle exocytosis in neurons and endocrine cells, homologous CAPS and Munc13 proteins play a similar role in mediating SNARE complex assembly for vesicle priming, however they may do so by distinct mechanisms. SNARE-binding in the CAPS and Munc13 proteins appears to reside within the CATCHR domain, which may also mediate additional protein interactions. CAPS and Munc13-1 collaborate with the SM protein Munc18-1 but the details of integration remain to be worked out. Studies are needed to determine whether accessory factors and SM proteins operate in concert or sequentially to assemble SNARE complexes, and to determine how these interactions occur within the confined space of juxtaposed membranes.

## Conflict of Interest Statement

The authors declare that the research was conducted in the absence of any commercial or financial relationships that could be construed as a potential conflict of interest.
